# Head Circumference and Neurocognitive Outcomes

**DOI:** 10.15844/pedneurbriefs-29-7-5

**Published:** 2015-07

**Authors:** J. Gordon Millichap

**Affiliations:** 1 Division of Neurology, Ann & Robert H. Lurie Children's Hospital of Chicago, Chicago, IL; 2 Departments of Pediatrics and Neurology, Northwestern University Feinberg School of Medicine, Chicago, IL

**Keywords:** Head Circumference, Wechsler Intelligence Scale for Children, Neurocognitive Disorders

## Abstract

Investigators from Universities of Glasgow and Bristol, UK, determined the value of head circumference (HC) as a screening measure, the incidence of head centile shifting, and the relationship between extremes of head size and later neurodevelopmental problems.

Investigators from Universities of Glasgow and Bristol, UK, determined the value of head circumference (HC) as a screening measure, the incidence of head centile shifting, and the relationship between extremes of head size and later neurodevelopmental problems. Data were obtained from the Avon Longitudinal Study of Parents and Children (ALSPAC), an ongoing prospective population-based study investigating the health and development of children in southwest England. There were 10,851 children with >2 HC measurements. HC was measured routinely at 2, 9, and 18 or 24 months and by researchers at ages 4, 8, 12, and 18 months. At each age, 2% to 3% of children had scores that were <-2 or >2 SDs below or above the mean. More than 15% children showed centile shifts, but less than one-third of these were sustained at subsequent measurements. Only 0.5% showed a sustained shift beyond the normal range. The WISC was used to measure IQ in research clinics at age 8 years for all. Neurocognitive disorders (NCDs) were identified from chart revue. Children with consistently small heads were up to 7 times more likely to have an NCD, but 85% of children with small heads had no NCD, and 93% of children with NCDs had head SD scores within the normal range.

HC centile shifts within the normal range are common and appear to reflect measurement error. Extreme head size is neither specific nor sensitive for detecting NCDs. Routine measurement of HC is unhelpful as a screening test or predictor of later developmental problems. [1]

COMMENTARY. Measurement of head circumference (HC) in children is prone to error for many reasons, including inexperience of the operator, lack of patient cooperation, and variability in hair growth and volume. The importance of the HC measurement as part of the neurologic examination warrants the personal attention of the neurologist. Technical errors excluded, a change from a normal baseline measurement at birth and 3-5 days to a microcephalic reading (<2nd percentile) or macrocephaly (>98th percentile) at 1 – 6 months would indicate a change necessitating neuroimaging: CT scan or MRI.

Microcephaly presents as primary or acquired [2]. Causes of primary microcephaly include autosomal dominant and autosomal recessive genetic disorders: trisomy 13, 18, and 21; Cornelia de Lange syndrome, Smith-Lemli-Opitz syndrome, and Rett syndrome, and hypothyroidism. Acquired microcephaly is characterized by a normal HC at birth, followed by microcephalic measurements in subsequent months or years, usually due to lack of brain development or growth. Causes of acquired microcephaly include sequelae from stroke, meningitis, encephalitis, toxoplasmosis, rubella, cytomegalovirus, and herpes; in utero teratogen exposure, and hypoxic-ischemic encephalopathy.

Macrocephaly, diagnosed by routine HC measurements during the first 10 months of life, is explained chiefly by hydrocephalus or cysts. A nationwide study of medical records of all Norwegian children <5 years of age hospitalized for intracranial expansion during a 4-year period found hydrocephalus was the primary diagnosis, affecting 173 (58%) of 298 patients; 57 (19%) had intracranial tumors and 68 (23%) had cysts and other primary diagnoses. Increased HC is important mainly in detecting hydrocephalus and cysts, especially in the first 10 months of life [3]. According to Wright et al [1], routine measurement of HC throughout infancy and early childhood is indicated only when an early measurement is outside +2 SDs. The recommendation of HC measurements routinely from birth to 24 months of age should be reconsidered.
